# Age- and Intravenous Methotrexate-Associated Leukoencephalopathy and Its Neurological Impact in Pediatric Patients with Lymphoblastic Leukemia

**DOI:** 10.3390/cancers13081939

**Published:** 2021-04-16

**Authors:** Ilona Rijmenams, Daan Moechars, Anne Uyttebroeck, Ahmed Radwan, Jeroen Blommaert, Sabine Deprez, Stefan Sunaert, Heidi Segers, Céline R. Gillebert, Jurgen Lemiere, Charlotte Sleurs

**Affiliations:** 1 Department of Brain and Cognition, KU Leuven, 3000 Leuven, Belgium; rijmenams_ilona@hotmail.com (I.R.); daan.moech@gmail.com (D.M.); celine.gillebert@kuleuven.be (C.R.G.); 2 Department of Pediatric Oncology, KU Leuven, 3000 Leuven, Belgium; anne.uyttebroeck@uzleuven.be (A.U.); heidi.segers@uzleuven.be (H.S.); 3 Department of Pediatric Hemato-Oncology, University Hospital Leuven, 3000 Leuven, Belgium; jurgen.lemiere@uzleuven.be; 4 Leuven Cancer Institute, KU Leuven, 3000 Leuven, Belgium; ahmed.radwan@kuleuven.be (A.R.); jeroen.blommaert@kuleuven.be (J.B.); sabine.deprez@kuleuven.be (S.D.); stefan.sunaert@uzleuven.be (S.S.); 5 Leuven Brain Institute, KU Leuven, 3000 Leuven, Belgium; 6 Department of Imaging and Pathology, KU Leuven, 3000 Leuven, Belgium; 7 Department of Gynaecological Oncology, KU Leuven, 3000 Leuven, Belgium

**Keywords:** childhood hematology, chemotherapy, neurotoxicity, leukoencephalopathy, risk classification

## Abstract

**Simple Summary:**

In this study, we investigated standardized post-chemotherapy magnetic resonance (MR) scans for leukoencephalopathy and patient- and treatment-related risk factors in childhood leukemia patients. As prevalence numbers are limited, our study provides the required estimations for this population. Furthermore, we demonstrate that younger patients might be more at-risk for development of leukoencephalopathy (LE), and that a higher intravenous methotrexate (IV-MTX) dose has a cumulative toxic effect, while the number of intrathecal administrations was not significantly associated with the extent of LE. This can suggest we should modify chemotherapeutic treatment regimens by decreasing the number of IV-MTX applications, with special attention for younger patients.

**Abstract:**

Methotrexate (MTX) is associated with leukoencephalopathy (LE) in children treated for lymphoblastic leukemia/lymphoma (ALL/LBL). However, large-scale studies with systematic MR acquisition and quantitative volumetric lesion information remain limited. Hence, the prevalence of lesion burdens and the potential risk factors of LE in this population are still inconclusive. FLAIR-MRI scans were acquired at the end of treatment in children who were treated for ALL/LBL, which were quantitatively analyzed for LE. Voxels were assigned to the lesion segmentation if indicated by two raters. Logistic and linear regression models were used to test whether lesion presence and size were predicted by risk factors such as age at diagnosis, gender, intrathecal (IT-) or intravenous (IV-)MTX dose, CNS invasion, and acute neurological events. Patients with a pre-existing neurological condition or low-quality MR scan were excluded from the analyses. Of the 129 patients, ten (8%) suffered from CNS invasion. Chemotherapy-associated neurological events were observed in 13 patients (10%) during therapy, and 68 patients (53%) showed LE post-treatment. LE was more frequent in cases of lower age and higher cumulative IV-MTX doses, while the extent of LE and neurological symptoms were associated only with IV-MTX doses. Neurological events were not significantly associated with LE, even though symptomatic patients demonstrated a higher ratio of LE (*n* = 9/13) than asymptomatic patients (*n* = 59/116). This study suggests leukoencephalopathy frequently occurs in both symptomatic and asymptomatic leukemia patients. Younger children and patients treated with higher cumulative IV-MTX doses might need more regular screening for early detection and follow-up of associated sequelae.

## 1. Introduction

Acute leukemia is the most frequent childhood malignancy, representing one-third of all childhood cancer diagnoses [1]. Over the last few decades, more effective multimodal chemotherapy regimens have greatly improved the outcome of children with acute lymphoblastic leukemia (ALL)/lymphoblastic lymphoma (LBL). As a result, the overall five-year survival rate has increased up to almost 90% [2]. Hence, long-term sequelae receive more and more attention. Traditionally, treatment through the 1980s included cranial irradiation. However, this treatment was associated with detrimental cognitive sequelae. Hence, the exclusion of cranial irradiation in contemporary treatments reduced such adverse side effects. However, the substitute treatment with intrathecal and systemic intravenous methotrexate (MTX) is not without risk. Even in the absence of cranial irradiation, childhood ALL survivors can still experience detectable deficits in specific neurocognitive functioning, particularly regarding processing speed and executive functions [3] such as organization/planning, cognitive flexibility, working memory, and verbal fluency [4]. Corresponding to these cognitive side effects, childhood cancer treatment also has the potential to induce adverse acute neurological symptoms such as seizures, headaches, sensory deficits, occlusive vascular-like events mimicking transient ischemic attacks, paresis, ataxia, visual abnormalities, altered mental status [5,6,7], and/or leukoencephalopathy (LE), which are most commonly detected as part of posterior reversible encephalopathy syndrome (PRES) [8].

However, the underlying neurotoxic mechanisms remain inconclusive. Such symptoms and LE could be a result of demyelination in leukemia patients treated with MTX. Given that the brain is highly vulnerable during the myelination process, altered myelination can have a greater long-term impact with reduced white matter volumes [9] and altered white matter microstructure [10] eventually resulting in decreased cognitive scores and academic delay [5,9,11].

The most frequently described image modality investigating white matter abnormality in ALL/LBL, is T2-weighted or Fluid-attenuated inversion recovery (FLAIR–)MR imaging to detect observable LE. Improved clinical awareness and improved imaging techniques have contributed to higher incidence rates [12]. As a consequence, LE is also increasingly detected in patients who do not show acute neurological events during treatment. Bhojwani et al. (2014) reported evidence of LE on at least one MRI-scan during treatment in 23.3% of their study sample (*n* = 369), of which 20.6% were asymptomatic (*n* = 355) and 92.9% were symptomatic patients (i.e., exhibiting neurological events) (*n* = 14) [13]. Hence, a higher incidence of LE in symptomatic vs. asymptomatic patients was demonstrated, albeit not statistically tested. After intensive CNS prophylactic treatment in ALL/LBL, LE can also become chronic in long-term survivors [14]. As reported recently [14,15], the prevalence rates of chronic LE ranged between 18% and 52% in cross-sectional ALL/LBL survivor studies with intervals of follow-up between 18 months and 15 years. In addition, neurocognitive deficits tended to be more prevalent in survivors with a history of LE, such as worse measures of organization and initiation [5], attention, processing speed and memory [16], and cognitive fluency [17]) or more acute seizures [18]. Therefore, LE could possibly be seen as a predictive indicator of subsequent cognitive impairment [14].

Although the number of large-sample studies is still limited, the potential risk factors of developing LE have increasingly received attention. These can include age at diagnosis, gender, central nervous system involvement, cancer diagnosis, types and intensity of the chemotherapy, therapy duration, etc. [5,14,17,19].

However, results remain inconclusive. For instance, Partap et al. (2019) described a younger age at diagnosis (<6 years of age) as a risk factor for the development of chronic LE [14], whereas Anastasopoulou et al. (2019) reported patients with older ages to be more at risk [8]. Besides age, gender can also modulate the neurodevelopmental pathways. With regard to the white matter, boys have a wider developmental frame than girls [20]. This could explain why some studies evidenced girls to be more vulnerable for treatment-related decreases in attention and information processing [7,21]. Whether LE during childhood cancer is associated with gender, however, has not been addressed so far. Additionally, chemotherapy-induced neurological symptoms [22] and LE [23], are more frequently detected when a higher number of intrathecal (IT-) MTX applications were administered, as compared to fewer applications [18,24]. Correspondingly, Bhojwani et al. (2014) revealed an increased risk of developing LE in cases of higher MTX levels at 42 h and more IT-MTX applications. In addition, Reddick et al. (2005) also encountered a higher prevalence of LE in patients treated with greater exposure to intravenous (IV-) MTX [24]. These studies suggest dose-related complications and long-term sequelae. However, such a dose-response relationship for developing LE cannot always be replicated [5,25,26]. In addition, studies did not always estimate the differential effects of higher doses or other risk factors. Such prediction models require a sufficiently large sample size. Finally, the quantitative information of exact lesion sizes has only been investigated in a limited manner to date [27].

More insight is needed regarding the exact prevalence and the specific risk factors for LE. Therefore, we retrospectively investigated LE and potential risk factors in ALL/LBL patients. We hypothesized that white matter lesions at the end of therapy are more likely to occur and are more extensive (a) in younger children in comparison with older children, (b) in females rather than in males, (c) in children treated with higher doses of CNS prophylaxis (i.e., IT-MTX applications and cumulative IV-MTX dose) and (d) in children with CNS invasion compared to children without CNS invasion. Finally, we hypothesized a higher risk of neurological symptoms in children with (a) more and (b) larger lesions than in children who did not show lesions.

## 2. Materials and Methods

### 2.1. Participants

All patients between 0 and 19 years old who were diagnosed with ALL/LBL between May 1999 and March 2017 and treated according to the EORTC-CLG 58951 protocol [28] or the guidelines of the EORTC-CLG 58081 study [29] (for treatment details, see Table S1) in the University Hospitals Leuven in Belgium were eligible, as they had completed the standard two-year treatment. All of these 171 patients received a standardized MR scan protocol at the end of treatment (i.e., 1.36–2.87 years after diagnosis) as part of the clinical procedure, which included a fluid-attenuated inversion recovery (FLAIR) MRI scan. Exclusion criteria consisted of a genetic syndrome (Down syndrome *n* = 4, Noonan syndrome *n* = 1), (pre-existing) neurological disease (intracranial thrombosis *n* = 4, ventricular drain *n* = 1, meningitis *n* = 1, spina bifida occulta *n* = 1, ataxia *n* = 1), vascular disease (vena cava superior syndrome *n* = 2), visually observed poor quality of the scan (motion artifacts: *n* = 25), high case complexity due to bone marrow transplant (*n* = 1), and no follow-up scan available (*n =* 1), which resulted in 129 included MR scans. Detailed information of the included cohort is provided in Table 1. This retrospective study was approved by the ethical committee of University Hospitals Leuven (S63052).

### 2.2. Data Acquisition

*Magnetic Resonance Imaging.* FLAIR MRI scans were acquired on 1T (*n* = 4), 1.5T (*n* = 69), and 3T (*n* = 56) MRI-scanners (Philips Achieva, Gyroscan NT, Ingenia and Intera and Siemens systems Aera, Magnetom Expert, Sonata Vision and Magnetom Symphony-Vision) at the University Hospital Leuven. Slice thickness varied between 3 to 6 mm, repetition time was 4–11 s, and echo time was 0.086–0.38 s. The resolutions of the images were between 391 mm × 391 mm × 575 mm and 1.000 mm × 1.016 mm × 7.500 mm.

*Imaging Preprocessing.* The preprocessing of the FLAIR scans was aimed at improving the manual and automatic detection of lesions. Images were denoised through the Advanced Normalization Tools (ANTsR v.0.4.9) denoise function [30] and corrected for bias fields through the N4 algorithm (ANTsR). Skull stripping was performed using HD-BET [31]. The resulting skull-stripped scans were resampled to 0.5 mm× 0.5 mm× 1 mm using ANTsR.

**Figure 1:**
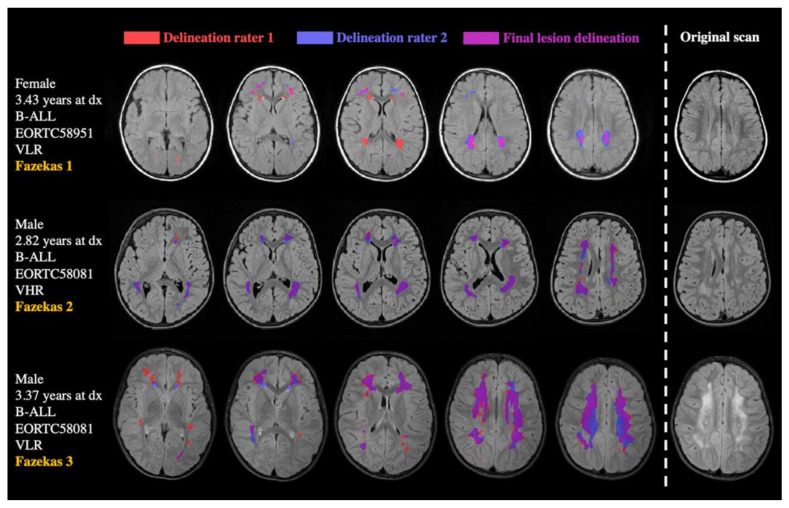
Examples of delineated lesions in three B-ALL cases. Cases are ordered by Fazekas rating (in yellow on the left), with Fazekas 1 showing the lowest lesion load in the upper case. Separate delineations of the two raters are presented in red and blue. The overlapping area, which was defined as final lesion mask, is depicted in pink.

*Lesion Masks.* Manual delineations of abnormal white matter intensities were performed in native space by two neuroscientist raters (2–6 years of experience) using ITK-SNAP segmentation software. Raters were trained and sample maps were validated by an experienced neuroradiologist (6 years of experience) [32]. Voxels were assigned to the final lesion mask if both raters indicated the voxel to be lesioned. Total lesion volumes were calculated for these overlapping areas (i.e., the number of voxels multiplied by the voxel size) (Figure 1), lesions were divided by intracranial volume (calculated by voxel size x number of voxels after skull-stripping [31], i.e., relative lesion volumes), and log-transformed due to their skewed distributions. The log-transformed values were used for the statistical analyses. As validity checks, (1) Pearson correlations between the numbers of delineated voxels of the two raters, (2) Spearman rank correlations between the Fazekas rating (performed by neuroradiologist A.R.) and the final relative lesion volume [33], and (3) the sensitivity and specificity of the final lesions were calculated (using the Fazekas rating as the gold standard).

*Medical Assessments.* Patient demographics were derived from medical records, recording: diagnosis, age at diagnosis, gender, treatment protocol, risk group (as defined by the treatment protocol), number of methotrexate injections (IT-MTX (6–12 mg/application) and IV-MTX (5 g/m^2^/application) (Table S1), CNS invasion, and neurological symptoms during therapy (i.e., epilepsy, paresis, transient ischemic attacks, and syncope).

### 2.3. Data Analyses

We hypothesized four factors to be associated with the occurrence and extent of lesions two years after diagnosis: age at diagnosis, gender, treatment intensity (as defined by the number of IT-MTX and IV-MTX applications), and CNS invasion. For treatment intensity, the number of intrathecal MTX applications and the cumulative intravenous MTX dose (5 g/m^2^ x applications) were implemented as two separate predictors (i.e., 5 predictors in total) (see Figure S1 for hypotheses and statistical models).

First, lesion presence (0/1) was predicted in a logistic regression model based on the 5 predictors of interest. This model was tested based on the complete database of included scans (*n* = 129). Second, a linear regression model was tested to predict the log-transformed relative lesion volumes (Figure S2), based on the same predictor set, in the subset of patients exhibiting lesions only. Finally, to test the categorical prediction of neurological symptoms (yes/no) based on lesion presence or lesion size, two separate logistic regressions were implemented (based on the entire database and patients with lesions only, respectively). The significant predictors of the first two models were used as covariates in the latter model to predict the neurological symptoms.

## 3. Results

*Lesion Prevalence.* Of 129 children diagnosed with ALL/LBL that were included, lesions were observed in 68 patients by both raters (i.e., with a minimum value of lesion mask > 0). The correlations between the lesion delineations of the two raters were *r* = 0.75 for all (*n* = 129) and *r* = 0.78 for lesioned patients (*n* = 68). Correlations between the overlapping lesion masks and the Fazekas ratings were *r* = 0.53 for all participants, and *r* = 0.47 for lesioned patients (*p* < 0.001). Manual delineations had a sensitivity = 0.64 and specificity = 0.77, using the Fazekas rating as the gold standard.

*Lesion Risk Factors*. Of the patients showing lesions, 5 (7.35%) had CNS invasion at diagnosis and 9 (13.24%) suffered from neurological events during therapy, while these numbers were 5 (8.20%) and 4 (6.56%) in the 61 non-lesioned patients, respectively.

A logistic regression revealed that a 1-year increase in age at diagnosis decreased the risk of developing a lesion by a factor of β = −0.19, with an odds ratio of 82 (*p* < 0.001) (Table 2; heatmap for distributions in Figure S3). In addition, with increasing cumulative IV-MTX treatment intensity, the risk of exhibiting a lesion increased.

**Figure 2:**
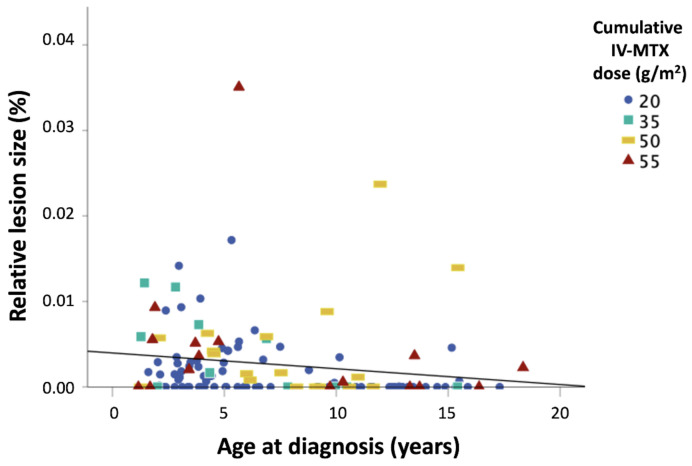
Scatter plot of lesion sizes and age at diagnosis. Individual ages at diagnosis are depicted (*y*-axis) against lesion size values (*x*-axis), colored by IV-MTX dose. Lower cumulative MTX doses of 20 g/m^2^ and 35 g/m^2^ are presented in dark and light blue, while higher doses of 50 and 55 g/m^2^ are presented in yellow and red, respectively. While the age range of non-lesioned patients (i.e., lesion size = 0.000) covers a wide range, the ages of lesioned patients are more densely distributed in the younger age range.

Based on the linear regression model testing the effects of risk factors on total lesion size, a positive association between cumulative IV-MTX dose (β = 0.31) and lesion size was observed (Table 3). A similar trend was observed for age at diagnosis, with younger patients being more at risk, but this finding was insignificant (β = −0.21, *p* = 0.10) (Figure 2).

**Figure 3:**
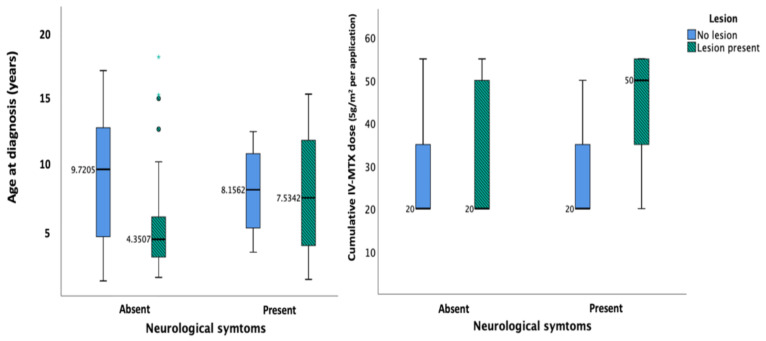
Box plots demonstrating the relationships between lesion presence, age, and cumulative IV-MTX dose. Median values of age at diagnosis (years) and cumulative IV-MTX dose are presented for subgroups of lesioned (green-striped) or non-lesioned (blue) patients, with or without neurological symptoms. Patients exhibiting lesions have lower ages at diagnosis on average (left panel). Both neurological symptoms and lesions are associated with higher cumulative IV-MTX dose (right panel).

*Neurological Symptoms*. Finally, to predict neurological symptoms (*n* = 13) (Table S2), the main predictors of lesion presence and size were added to two separate logistic models with IV-MTX as a covariate, based on its significance in the previous models (Table S3; Figure 3). These models demonstrated the significant effect of cumulative IV-MTX dose on the occurrence of neurological symptoms (*p* < 0.05) (heatmap in Figure S4), while symptoms were not significantly associated with lesion presence or size.

## 4. Discussion

The purpose of this retrospective MRI study was to investigate the prevalence and severity of leukoencephalopathy after chemotherapy-only-treatment in childhood leukemia patients, including possible risk factors, and its association with acute neurological events. We concluded that an increased risk of developing a lesion exists in younger patients, and patients treated with higher IV-MTX intensity. Additionally, the extent of the lesions and acute neurological events were associated with IV-MTX dose, but not with the occurrence of the lesions itself.

### 4.1. Prevalence and Severity of Lesions

In this retrospective MRI study, all eligible children diagnosed with ALL/LBL were included, regardless of neurological symptoms during therapy. We found LE in 68/129 (53%) patients, which is equivalent to several previous studies which obtained standardized MR imaging after treatment [15,18,24]. By contrast, studies which only implemented MR imaging after acute neurological events demonstrated much higher LE prevalence numbers [12,34,35]. Lower risk ratios for LE were encountered in cases of lower chemotherapy intensity [13], or at longer times of follow-up [17,25,26,36]. Hence, an interpretation of prevalence numbers across studies requires caution. In this study, all available data of standardized post-treatment MR-scans were analyzed retrospectively. Since the minority of patients treated for ALL/LBL experience acute neurological symptoms, we aimed to investigate the occurrence of LE independently from symptoms. This can help increase awareness for the standard follow-up of asymptomatic patients as well.

In spite of the fact that symptoms and radiological findings appear to normalize in most children, they can persist and become chronic in some cases [14]. Studies investigating chronic LE demonstrated that about 70%–78% of the subsample of patients who developed LE during chemotherapy still show LE at least three years later [5,25]. Although chemotherapy is assumed to induce fewer neurological side effects than cranial RT, these odds of developing LE thus still seem relatively stable in contemporary treatments [14].

### 4.2. Risk Factors for Leukoencephalopathy/Neurotoxicity

First, gender did not affect the incidence and/or size of LE. This result confirms earlier findings [5,26], with observable LE being equally distributed between sexes [16].

On the other hand, Atra et al. (2004) observed that 95% of the children who developed neurological symptoms during treatment were boys, whereas some neurocognitive studies recognized female gender as a risk factor for neuropsychological decline [21,37,38,39]. This could suggest that gender-specific microstructural or myelination pathways might interact differently with induced neurotoxic mechanisms, resulting in different functional outcomes [22].

Second, younger age at diagnosis appeared to be an important risk factor for the absolute risk (occurrence) of lesions, which could be the result of early demyelination. Most white matter areas complete their myelination process before the age of 4 years [40,41], while the peak age of our cohort was between 2–4 years at diagnosis. Hence, this population might be specifically vulnerable to development of acute LE. This age effect was less strong for the size of the lesion. In other words, the risk of a neurotoxic hit might be higher in younger patients, while the cumulative treatment effects could result in an expansion of the lesions. Multiple previous studies also demonstrated younger children to have an increased risk of developing chronic LE [14,42,43] or cognitive decline [21,44,45,46]. On the other hand, some authors have declared age not to be a risk factor [7,23], or that higher age is a risk factor (>10 years) [8]. This contradiction could be the result of th interplay between multiple factors, such as the affected cell type, other toxicities, or heterogeneous treatments.

Third, our study indicated a significant contribution of cumulative IV-MTX dose to the risk of developing LE, as well as its extent, whereas no additional effect was found for IT-MTX applications. In previous studies, more cases showed LE if they were treated more intensively [13,23,24]. However, this was not always replicated [5,25], which could be explained by a thresholding effect. In addition, most previous studies did not delineate lesions [24], or did not differentiate between IT- and IV-MTX effects [13,18,22,23]. Only one previous study demonstrated similar quantitative effects of IV-MTX doses on lesion sizes [27]. The fact that IV-MTX, but not IT-MTX, was associated with lesions, might be explained by the higher dose per application (i.e., 5 g/m^2^ and 12 mg, resp.) and, consequently, high plasma concentrations, which can affect the brain microstructure through multiple toxic mechanisms [47]. We also refer to our previous findings of chronic LE in long-term osteosarcoma survivors who received high-dose IV-MTX without CNS-directed treatment [48]. This might imply that additional IT-MTX applications such as CNS prophylaxis could be applied without additional risk for LE toxicity.

In addition to LE, neurological symptoms were also associated with higher IV-MTX dose in our study, confirming the findings of Nassar et al. (2017) [18]. We found neurological symptoms in 13 out of 129 (10.08%) patients, of whom eight (42.85%) experienced seizures during therapy. A child who showed observable white matter hyperintensities was 1.825 times more likely to also suffer from a neurological complication, albeit this association was insignificant. More in-depth neuropsychological outcomes might be more sensitive for LE than for acute neurological events. Hence, the prolonged neurocognitive follow-up of patients is recommended in clinical practice.

### 4.3. Limitations

Several limitations should be discussed. First, the delineations were drawn manually, so they depended on the visual estimation of the rater. To be sufficiently stringent, we considered a voxel to be lesioned only if it was marked by 2 raters. This should greatly limit the influence of errors by any individual rater. Furthermore, the images were rated independently according, to the Fazekas rating (without any knowledge on the patient or image data) by the neuroradiologist as validity check. We should note that a post-hoc analysis predicting the lesion occurrence or extent based on Fazekas rating only, did not show any significant findings, which suggests manual delineations (with inter-rater correction) to be more sensitive, albeit very labor-intensive. Although an automatic lesion prediction algorithm was initially tested, this procedure did not stand up to expert scrutiny.

Second, our study did not specify in which phase of therapy neurological the symptoms were observed. As a consequence, no causal relationships can be estimated between cumulative doses and symptoms. In addition, the number of patients exhibiting neurological symptoms and CNS invasion were rather small (*n* = 13, 10, respectively), and the symptoms might have occurred during treatment, often shortly (within days) after an IT-MTX application. Only post-treatment scans were investigated in this study, on which the acute imaging anomalies might already have been resolved [49]. The lack of a significant association between LE, neurological symptoms and CNS invasion should thus be interpreted with caution.

In post-hoc checks for interdependency between any combination of two predictors, CNS invasion appeared to be associated with IV-MTX dose. Still, after the exclusion of the CNS invasion predictor from the model, the same significant findings were found. Furthermore, the cumulative corticosteroid dose (consolidation–maintenance phase) and IT-MTX applications appeared to be highly intercorrelated (*r* = 0.790), with an increase in the variance inflation factor (VIF) of IT-MTX if corticosteroid dose was added to the full model. Therefore, the final models did not include corticosteroids as an independent predictor in the current study, as we focused on the number of intrathecal MTX applications and intravenous MTX doses, given their CNS-directed application and high doses (i.e., 5 g/m^2^ in each administration), respectively. Nevertheless, in post-hoc analyses we also tested the full models including corticosteroid dose (instead of IT-MTX dose) as an independent predictor. Based on these models, exactly the same significance was found for age at diagnosis and IV-MTX dose as predictors. So, no corticosteroid effects were encountered in this study. Nevertheless, given that recent studies also provide evidence of the possible toxic effects of glucocorticosteroids on the cognitive outcomes [50] and grey matter [51] of leukemia patients, corticosteroid mechanisms should receive attention in future studies for populations with less interdependency between corticosteroids and chemotherapeutic agents. In addition, since the agent dosimetry was corrected for age as it was presented as cumulative dose per m^2^, or age-dependent (e.g., IT-MTX dose). No treatment–age interaction terms were included as predictors. Also, given that an IT-IV-MTX interaction term showed high interdependency (with a high VIF) with the other predictors, this interaction term was not possible to test using the current dataset. The interaction effects can be investigated more thoroughly in future studies in which specific agent doses are less interdependent and age-corrected to determine whether the encountered IV-MTX-induced neurotoxic effects depend also on other demographic or treatment-related factors.

Finally, the images used in this study were acquired over a longer period of time and on different MR-scanners. Given that the image quality differed greatly, the scans were screened by all raters to meet a certain standard of quality that ensured the rater’s ability to accurately segment the lesions. Only scans which all raters independently considered to be of sufficient quality were included in the analyses. Post-hoc analyses checks including the magnetic field strength as covariate showed no significant effect of the field strength, nor did this change any of the results. In this study, image intensity was only used for denoising, bias field correction, and visual delineations, but it was not implemented for any other processing steps or analyses. However, more and more applications applying artificial intelligence for lesion delineation nowadays make in-depth use of relative intensities [52], which should also be increasingly validated in future studies of leukemia.

### 4.4. Future Directions: Mechanisms and Long-Term Outcomes

It is possible to reverse a syndrome, e.g., in case of PRES, through lowering the blood pressure and reducing the eliciting medication. Delaying the next course of high-dose intravenous MTX might provide for the more efficient clearance of this neurotoxic agent [3,12,22]. Regardless of this reversibility, brain abnormalities have the potential to hinder brain maturation, which could induce permanent brain damage. The number of neuroimaging studies in survivors of leukemia has increased in the most recent years [10,53,54]. Only two investigated the link between microstructural brain changes and acute LE [5,25], showing LE to be associated with poorer white matter microstructure on DTI. The latter was more strongly related to neurocognitive function than LE, suggesting DTI to be a sensitive neurocognitive predictor. Whether specific tracts are more vulnerable for toxic hits and LE, remains a question for future studies. As the observable disruption of healthy brain development (e.g., LE) can result in neuroanatomical and functional changes, the early detection of LE might thus be of great importance [4,8]. Although the current data did not enable us to analyze the lesion locations in a voxel-wise manner, one can infer from a group-estimated heatmap after non-linear registrations to a pediatric template [55] (Figure S5), that lesions mostly occurred in the periventricular area, extending to a small extent to the juxtacortical areas. Multiple white matter tracts are present in these areas, with the highest lesion frequencies occurring in voxels covering the superior and inferior longitudinal fasciculus, inferior fronto-occipital fasciculus, and cingulum, but lesions also occurred in the corticospinal tract, uncinate fasciculus, corpus callosum, thalamic radiations etc.

Besides the MTX-dose-related effects on it [4], LE might also be modulated by multiple other factors, including antineoplastic drugs, arterial hypertension, renal dysfunction, endocrine, and epigenetics [8,12]. Physiological biomarker studies should receive attention as well regarding elevated CSF-extracted measures and worse cognitive outcomes, such as homocysteine levels [13,26], oxidated phosphatidylcholine [56], phosphorylated tau [57], and total Tau [58,59]. One study showed glial fibrillary acidic protein, myelin basic protein, and total tau to be significant predictors of LE [60]. Also, increased inflammatory markers were evidenced during ALL/LBL treatment [61], but have not yet been investigated with LE.

Future neuroimaging studies implementing physiological biomarkers will be required to unravel the exact neurotoxic mechanisms of each of these contributing factors, which could lead to LE and neurocognitive decline.

## 5. Conclusions

Given the increasing survival rates in leukemia, treatment-related long-term side effects are becoming increasingly important to address. This retrospective MRI study demonstrated evidence for the occurrence of LE during treatment in approximately 52% of cases, with younger aged patients being at increased risk. Furthermore, increased intravenous MTX intensity could potentially increase the risk of such lesions, as well as leading to expansion of the lesions and acute neurological symptoms (primarily seizures). Further research will be needed to better understand the neurotoxic mechanisms induced by ALL/LBL-specific chemotherapeutic protocols (including IT- and IV-MTX), its long-term neuro(psycho)logical impact, and how to minimize the possible deficits.

## Figures and Tables

**Table 1:** Patient characteristics.

	Number of Patients
Total sample	129
Gender	
Female (*n*) (%)	59 (46%)
Male (*n*) (%)	70 (54%)
Age at diagnosis (years)	
Median	5.65
Mean	7.18
Range	1.16–18.35
Treatment risk group ^A^	
Low (*n*)	20
Standard/high	
AR1 (*n*)	65
AR2 (*n*)	28
VHR (*n*)	16
Neurological symptoms ^B^ (*n*)	13
CNS invasion (*n*)	10

Notes: ^A^ Very low risk (VLR) group, standard/high risk is defined as average 1 (AR1), average 2 (AR2) and very high risk (VHR) group. ^B^ Defined as epilepsy, paresis, and transient ischemic attacks.

**Table 2:** Logistic regression analysis of effects of age, gender, treatment intensity, and CNS invasion on lesion prevalence.

Variable	*β*_1_ Coefficient	SE	*p* Value	Odds-Ratio	Chi-Square, *p* (Model)
Age (years)	−0.194	0.048	<0.001 ***	0.824	*χ*^2^ = 24.753
Gender	−0.306	0.393	0.436	0.736	*p* < 0.001 ***
Intrathecal MTX	−0.072	0.083	0.388	0.931	
Intravenous MTX	0.211	0.084	0.012 *	1.235	
CNS invasion	−0.927	0.789	0.241	0.396	

For intrathecal MTX, the number of intrathecal MTX applications was implemented as a predictor. The cumulative intravenous dose was used for intravenous MTX (5 g/m^2^ × number of applications). * indicates *p* < 0.05, *** indicates *p* < 0.001.

**Table 3:** Univariate linear regression analysis of effects of age, gender, treatment intensity, and CNS invasion on lesion size (logarithmically scaled).

Variable	*β*_1_ Coefficient	*t* Value	*p* Value	R^2^, F, *p* (model)
Age (years)	−0.207	−1.679	0.098	R^2^ = 0.119, *F* = 1.678, *p* = 0.153
Gender	0.132	1.092	0.279	
Intrathecal MTX	−0.021	−0.162	0.872	
Intravenous MTX	0.308	2.194	0.032 *	
CNS invasion	−0.019	−0.152	0.880	

For intrathecal MTX, the number of intrathecal MTX applications was implemented as a predictor. The cumulative intravenous dose was used for intravenous MTX (5 g/m^2^ × number of applications). * indicates *p* < 0.05.

## Data Availability

Anonymized datasets including non-identifiable data are available upon request.

## Supplementary Materials

The following are available online at https://www.mdpi.com/article/10.3390/cancers13081939/s1, Figure S1: Visual overview of hypotheses. Figure S2: Logarithmic transformation of relative lesion sizes. Figure S3: Heatmap of lesion occurrence by age at diagnosis and cumulative IV-MTX dose. Figure S4: Heatmap of neurological symptoms by age at diagnosis and cumulative IV-MTX dose. Figure S5: Heatmap of white matter lesions at group level mapped on a pediatric atlas. Table S1: Number of intravenous and intrathecal methotrexate applications in each risk group and EORTC-CLG protocol. Table S2: Characteristics of patients showing neurological symptoms during therapy. Table S3: Logistic regression analyses of effect of intravenous MTX and lesion presence/size on neurological symptoms
