# Supplementary material for: Age- and Intravenous Methotrexate-Associated Leukoencephalopathy and Its Neurological Impact in Pediatric Patients with Lymphoblastic Leukemia
Source: Cancers (Basel). 2021 Apr 16;13(8):1939. doi: 10.3390/cancers13081939 (PMC8073318; doi:10.3390/cancers13081939)
Supplement: Supplementary file 1 [file cancers-13-01939-s001.zip › cancers-1160568-supplementary.pdf]

Supplementary Materials

Age- and Intravenous Methotrexate-Associated Leukoencephalopathy and Its Neurological Impact in Pediatric Patients with Lymphoblastic Leukemia

Ilona Rijmenams [1,2,†], Daan Moechars [1,2,†], Anne Uyttebroeck [2,3,7], Ahmed Radwan [4,6,7], Jeroen Blommaert [5,6,7], Sabine Deprez [4,6,7], Stefan Sunaert [4,6,7], Heidi Segers [2,3,7], Céline R. Gillebert [1,6], Jurgen Lemiere [3,6,7] and Charlotte Sleurs [2,6,7]*

Hypothesis one

Age at diagnosis, gender, treatment intensity and CNS invasion influences the lesion presence.

**Model 1**	Age at diagnosis	→	Lesion presence
	Gender	→	
	Treatment intensity	→	
	CNS invasion	→	

Statistical model: logistic regression model

Hypothesis two

Age at diagnosis, gender, treatment intensity and CNS invasion influences the frequency the lesion size.

**Model 2**	Age at diagnosis	→	Lesion size
	Gender	→	
	Treatment intensity	→	
	CNS invasion	→	

Statistical model: univariate linear regression model

Hypothesis three

The presence of white matter lesion and white matter lesion size influences the prevalence of neurological symptoms during treatment.

**Model 3**	Lesion presence (+ significant covariates)	→	Prevalence of neurological symptoms during treatment

Statistical model: univariate logistic regression model

**Model 4**	Lesion size (+ significant covariates)	→	Prevalence of neurological symptoms during treatment

Statistical model: univariate logistic regression model

Figure S1. Visual overview of hypotheses.

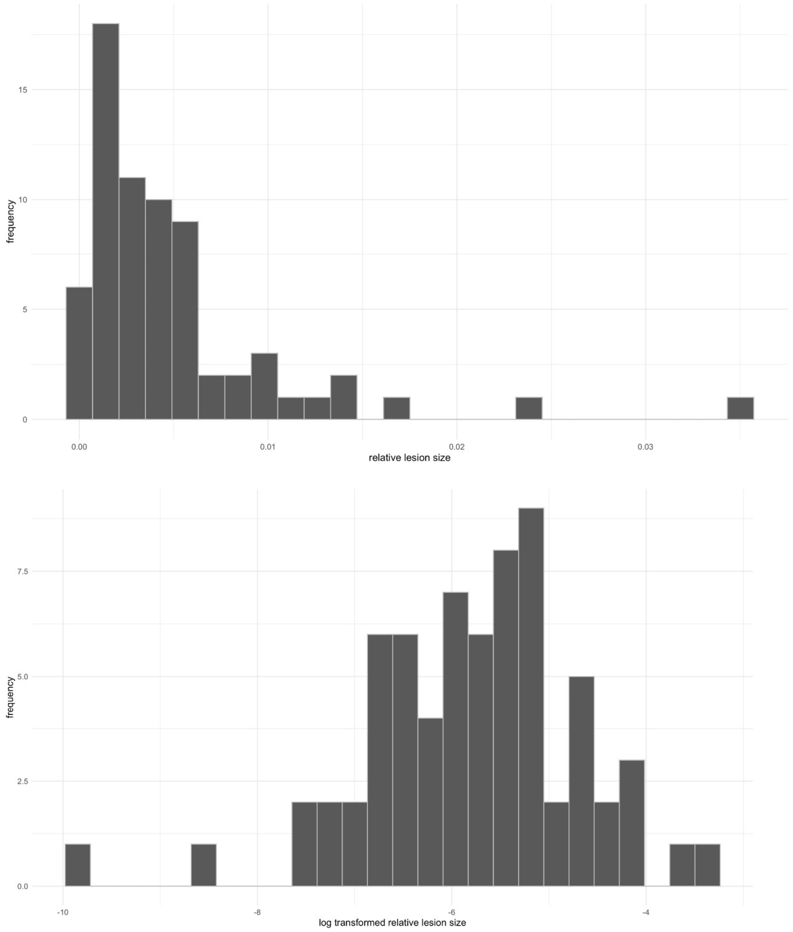

Figure S2. Logarithmic transformation of relative lesion sizes

Cum. IV-MTXdose	Age (years)															
	**<2**	**2**	**3**	**4**	**5**	**6**	**7**	**8**	**9**	**10**	**11**	**12**	**13**	**14**	**15**	**18yrs**
**20g/m²**	1/2	10/14	8/13	7/8	5/10	2/4	1/2	1/1	1/5	1/2	0/4	1/7	0/3	0/2	2/5	
**35g/m²**	2/2	1/2	1/1	1/2		1/1	0/1		0/1	0/1					0/1	
**50g/m²**	0/1	1/1		3/3	1/1	2/2	1/1	0/1	1/3	1/2	1/2				1/1	
**55 g/m²**	2/4		3/3	1/1	1/1				0/1	1/1			1/3			1/1

Figure S3. Heatmap of lesion occurrence by age at diagnosis and cumulative IV-MTX dose. Note. Age is categorized by each year (e.g. 2years represents the age category of 2-3years old). For each cell, the relative percentage of patients showing a lesion is presented (e.g. out of 2 patients of <2years old receiving 20g/m², 1 patient had a lesion). Cumulative MTX doses are presented, depending on the treatment protocol and the patient's risk group (with each separate administration of 5g/m²). Low percentages are presented in green, higher percentages in orange and red. Both age and IV-MTX dose appear associated with the risk of developing lesions.

Cum. IV-MTXdose	Age (years)															
	**<2**	**2**	**3**	**4**	**5**	**6**	**7**	**8**	**9**	**10**	**11**	**12**	**13**	**14**	**15**	**18yrs**
**20g/m²**	0/2	1/14	/13	/8	0/10	/4	1/2	0/1	0/5	0/2	0/4	1/7	0/3	0/2	1/5	
**35g/m²**	1/2	0/2	0/1	0/2		0/1	0/1		0/1	0/1					0/1	
**50g/m²**	0/1	0/1		0/3	0/1	0/2	1/1	0/1	1/3	1/2	1/2				0/1	
**55g/m²**	0/4		1/3	0/1	1/1				0/1	0/1			1/3			0/1

Figure S4. Heatmap of neurological symptoms by age at diagnosis and cumulative IV-MTX dose. Note. Age is categorized by each year (e.g. 2years represents the age category of 2-3years old). For each cell, the relative percentage of patients showing a neurological symptom is presented (e.g. out of 2 patients of <2years old receiving 35g/m², 1 patient had a neurological symptom). Cumulative MTX doses are presented, depending on the treatment protocol and the patient's risk group (with each separate administration of 5g/m²). Low percentages are presented in green, higher percentages in orange and red. Across ages, IV-MTX dose is associated with the risk of developing neurological symptoms.

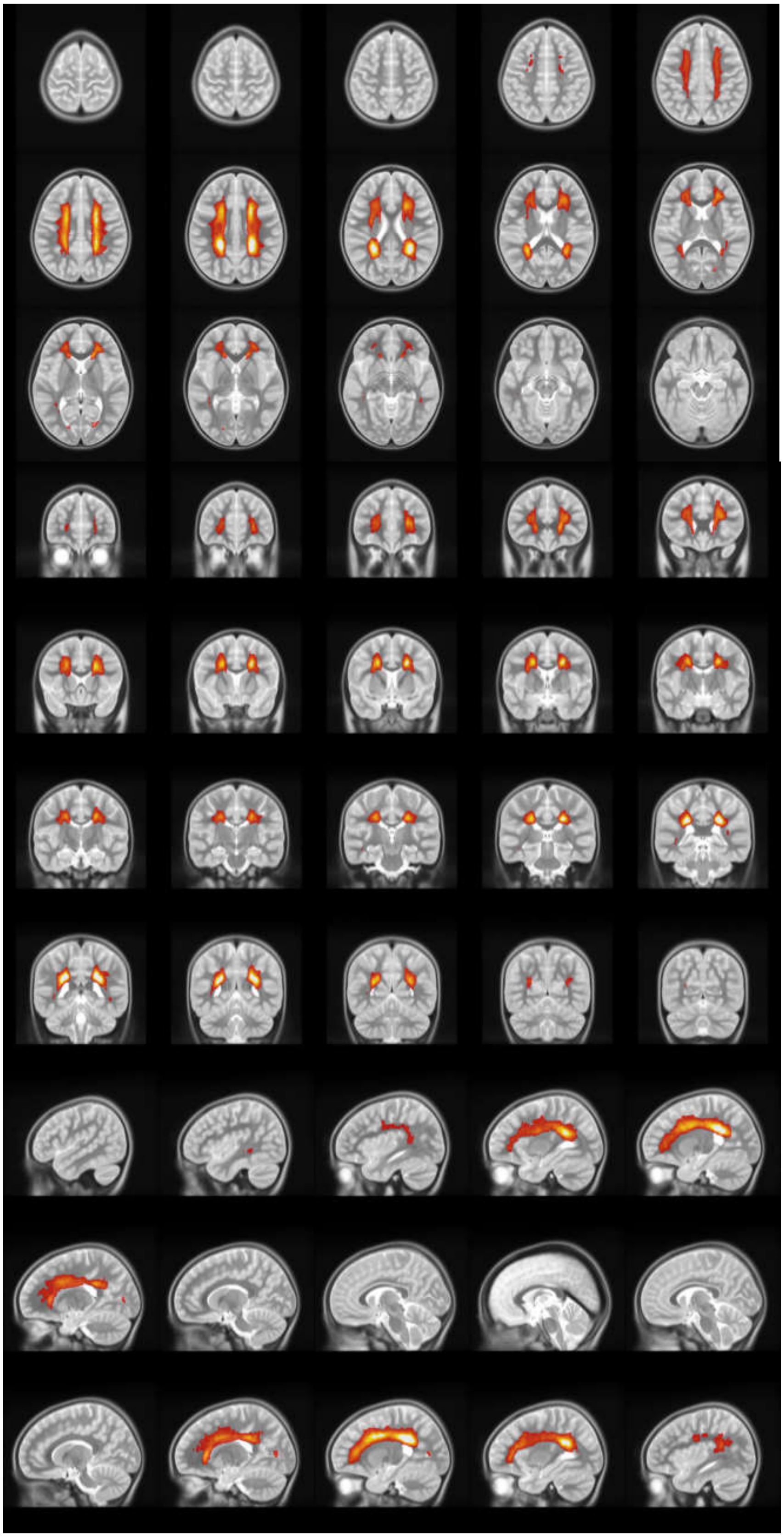

Figure S5. Heatmap of white matter lesions at group level mapped on a pediatric atlas. Note. Based on individual binary lesion maps that were nonlinearly co-registered do a pediatric template

(Fonov et al. 2011), one average lesion map across subjects in this pediatric template space was calculated and depicted.

Table S1. Number of intravenous and intrathecal methotrexate applications in each risk group and EORTC-CLG protocol

		EORTC 58081	EORTC 58951	EORTC 58081	EORTC 58951	EORTC 58081	EORTC 58951	EORTC 58081	EORTC 58951
		**VLR**	**VLR**	**AR1**	**AR1**	**AR2 B/T**	**AR2**	**VHR**	**VHR**
Prephase	**IV-MTX**								
	**IT-MTX**	1	1	1	1	1/1	1	1	1
Induction (IA)	**IV-MTX**					1/0	1		1
	**IT-MTX**	2	2	2*	2*	2/2*	2*	2*	2*
Consolidation (IB, IB'+VANDA)	**IV-MTX**							1	2
	**IT-MTX**	2	2	2*	2*	2/2*	2*	4*	3*
Interval	**IV-MTX**	4	4	4	4	4/4*	4	6	3
	**IT-MTX**	4	4*	4*	4*	4/4*	4*	6*	3*
Intensification (IIA + IIB, R1-3x2)	**IV-MTX**								4
	**IT-MTX**	2	1	2*	1*	4/2*	1*	4*	6*
Maintenance	**IV-MTX**					6/6	6		
	**IT-MTX**			6*	6*	6/6*	6*		5*
Total	**IV-MTX**	**4**	**4**	**4**	**4**	**11/10**	**11**	7	**10**
	**IT-MTX**	**11**	**10**	**17**	**16**	**19/17**	**16**	17	**20**

Note. * indicates triple chemotherapy (i.e. IT-MTX was administered with ARA-C and hydrocortisone). IT-MTX=intrathecal methotrexate. IV-MTX=intravenous methotrexate. IT-MTX dose per application = 6mg (patient <1year old), 8mg (>1<2 years), 10mg (>2<3 years) 12mg (>3 years). IV-MTX dose per application = 5g/m^2.

Table S2. Characteristics of patients showing neurological symptoms during therapy

	**Age at dx**	**Gender**	**Diagnosis**	**Risk group**	**Protocol**	**CNS invasion**	**Neurological symptoms**	**Lesion size (mm^3)**
01.	7.068	Female	B-ALL	AR1	EORTC 58951	No	Epilepsy	0
02.	15.496	Female	B-ALL	VLR	EORTC 58951	No	Paresis & TIA	933.4996
03.	5.655	Female	B-ALL	AR2	EORTC 58951	No	Epilepsy	47712.77
04.	11.962	Female	T-ALL	AR2	EORTC 58081	No	Syncopes	32320.25
05.	2.781	Male	B-ALL	AR1	EORTC 58951	No	Epilepsy	2140.065
06.	3.866	Female	B-ALL	AR2	EORTC 58951	Yes	Paresis & TIA	4881.881

07.	10.973	Female	B-ALL	VHR	EORTC 58951	No	Epilepsy	1717.378
08.	3.367	Female	B-ALL	AR1	EORTC 58951	No	Epilepsy	0
09.	12.622	Female	B-ALL	AR1	EORTC 58951	No	Paresis	0
10.	13.499	Male	T-ALL	AR2	EORTC 58951	Yes	Epilepsy	5499.392
11.	1.274	Male	B-ALL	VHR	EORTC 58081	No	TIA	7186.134
12.	7.534	Female	B-ALL	VHR	EORTC 58951	No	Epilepsy	2465.969
13.	9.244	Male	T-ALL	AR2	EORTC 58081	No	Epilepsy	0

Table S3. Logistic regression analyses of effect of intravenous MTX and lesion presence/size on neurological symptoms

Variable	β_1 coefficient	SE	P Value	Odds-ratio	Chi-square, p (model)
**Intravenous MTX**	.201	.097	.038	1.223	χ^2=5.915, *p*=.052
**Lesion presence**	.602	.644	.350	1.825	
**Intravenous MTX**	.297	.131	.024	1.346	χ^2=6.032, *p*=.049
**Lesion size**	-.037	.846	.965	.964	
